# Is asparaginase encapsulated in erythrocytes effective as second‐line treatment in acute lymphoblastic leukaemia?

**DOI:** 10.1111/bjh.18372

**Published:** 2022-07-20

**Authors:** Inge M. van der Sluis, Yves Bertrand, André Baruchel, Rob Pieters

**Affiliations:** ^1^ Princess Máxima Center for Pediatric Oncology Utrecht The Netherlands; ^2^ Insitute d'Hématology et d'Oncologie Pediatric (IHOPE) Pediatric Hematology and Immunology Lyon France; ^3^ Hôpital Universitaire Robert Debré (APHP) Hemato‐Immunology/Pediatric Hemato‐immunology Paris France

To the Editor,

With interest, we have read the recent article by Lynggaard and colleagues.^1^ They studied a new formulation of asparaginase encapsulated in erythrocytes (Eryaspase) in acute lymphoblastic leukaemia (ALL) patients with a prior hypersensitivity reaction to pegylated *E. coli* asparaginase. Eryaspase was well‐tolerated and most patients had therapeutic asparaginase levels after the first infusion. Based on this study, the US Food and Drug Administration granted fast‐track designation to Eryaspase for ALL patients with hypersensitivity reaction to pegylated asparaginase. However, in our opinion, the efficacy of Eryaspase in this patient group remains in fact questionable.

To begin, the pharmacodynamic goal of asparaginase therapy is asparagine depletion, and unfortunately this was not measured. It is well known that asparagine cannot be measured reliably with free asparaginase in the serum, due to the continuing breakdown of asparagine after sampling.^2^ Therefore, serum asparaginase activity is used as a surrogate marker for different asparaginase preparations used currently, such as native *E. coli* asparaginase, PEGasparaginase and *Erwinia* asparaginase.^3^ However, when asparaginase is encapsulated in the erythrocytes, breakdown of asparagine takes place inside the erythrocytes and therefore serum asparagine level can be measured more reliably. The feasibility of asparagine measurement after Eryaspase has been demonstrated in previous studies. Domenech *et al*.^4^ treated relapsed/refractory ALL patients without prior hypersensitivity reactions to asparaginase with Eryaspase and serum asparagine was not depleted in two out of 18 patients. In elderly ALL patients without prior hypersensitivity reactions asparagine was not depleted in 15%–29% of the patients seven days after Eryaspase administration.^5^ In patients with a prior hypersensitivity reaction only a 50% reduction in plasma asparagine concentration was found three days following Eryaspase infusion, which was followed by a slow return towards baseline before the next infusion.^6^ The number of patients without asparagine depletion could in fact be high in Lynggaard *et al*.'s study as well, as only patients with a prior hypersensitivity reaction were included. Even with technical issues caused by lysis of red blood cells resulting in free asparaginase in the serum or plasma after sampling, measurable asparagine levels would still be very informative indicating insufficient asparaginase activity.

Secondly, the authors did measure asparagine in the cerebrospinal fluid (CSF) and found a mild decline after administration of Eryaspase. Asparagine levels remained rather high compared to levels found after treatment with native *E. coli* asparaginase, *Erwinia* asparaginase and even PEGasparaginase.^7^, ^8^, ^9^, ^10^ CSF asparagine levels are depleted in part by continuous exchange of asparagine between serum and CSF pools.^11^, ^12^ In addition, *E. coli* asparaginase and *Erwinia* asparaginase can deplete the CSF in almost all patients, probably due to small amounts of asparaginase entering the central nervous system, whereas the bigger PEGasparaginase cannot.^12^ Similar to PEGasparaginase, Eryaspase's efficacy will depend on the exchange of asparagine between serum and CSF. However, PEGasparaginase showed a better, deeper CSF asparagine depletion compared to Eryaspase.^9^, ^10^ This might suggest less serum asparagine depletion and less efficacy of Eryaspase.

Thirdly, serum or plasma activity levels of non‐encapsulated asparaginase above 100 IU/L are considered to be therapeutic and are associated with complete asparagine depletion.^3^ To measure asparaginase activity levels of Eryaspase, lysis of the erythrocytes is needed. It is unknown whether this whole‐blood activity level after lysis of red blood cells can be compared one‐on‐one with serum/plasma levels of non‐encapsulated formulations. Can the same cut‐off of activity levels above 100 IU/L be used to define therapeutic activity levels of Eryaspase? The authors stated themselves that this method needs validation. Additionally, the interpretation of the results needs further validation.

Fourthly, 60% of the patients developed anti‐asparaginase antibodies during Eryaspase treatment, which seems remarkably high as asparaginase is hidden in the red blood cells. A fraction of free asparaginase is needed to trigger the development of anti‐asparaginase antibodies. Besides causing clinical allergic reactions, these antibodies also can accelerate asparaginase clearance. Lynggaard and colleagues describe that these antibodies cannot penetrate the erythrocyte membrane and may not target the encapsulated asparaginase. After the first Eryaspase infusion 96.1% of the patients had trough asparaginase activity levels above 100 IU/L. This had dropped to 66.7% after the fourth infusion in the two‐week schedule, which was attributed to small numbers (nine patients). Nevertheless, the same tendency was found in 37 patients in the six‐week schedule, with only 72% of 151 through samples being above 100 IU/L. Thus, regardless of whether or not whole‐blood asparaginase activity levels are reliable, trough activity levels do decrease after repeated administrations, which might be due to developing antibodies. These data suggest insufficient activity in approximately one third of the patients following repeated administrations.

In conclusion, it would be of utmost importance that Lynggaard and colleagues add serum asparagine levels to the report. We do believe that measurement of asparagine is feasible in this peculiar context and is absolutely necessary to determine the efficacy of Eryaspase in ALL patients, which is different from that of other asparaginase preparations, especially in those with a prior hypersensitivity reaction to asparaginase. Also, the efficacy of repeated administrations needs further evaluation. Given the caveats of the asparaginase activity levels with this specific preparation, and the lack of information on asparagine depletion, we cannot conclude from this study that Eryaspase is efficacious.

## CONFLICT OF INTEREST

Inge M. van der Sluis: research support, advisory board, speaker fees from Jazz Pharmaceuticals, Servier, Clinigen. Yves Bertrand: advisory board Jazz Pharmaceuticals and Servier; research support Erytech. André Baruchel and Rob Pieters: research support, advisory board, speakers fees from Jazz Pharmaceuticals, Servier, Clinigen and Erytech.
